# Temporal Characteristics of Visual Processing in Amblyopia

**DOI:** 10.3389/fnins.2021.673491

**Published:** 2021-06-03

**Authors:** Xia Hu, Yi Qin, Xiaoxiao Ying, Junli Yuan, Rong Cui, Xiaowei Ruan, Xianghang He, Zhong-Lin Lu, Fan Lu, Fang Hou

**Affiliations:** ^1^School of Ophthalmology & Optometry and Eye Hospital, Wenzhou Medical University, Wenzhou, China; ^2^Biosysen Ltd., Shenzhen, China; ^3^Fuzhou Aier Eye Hospital, Fuzhou, China; ^4^Division of Arts and Sciences, NYU Shanghai, Shanghai, China; ^5^Department of Psychology, Center for Neural Science, New York University, New York, NY, United States; ^6^NYU-ECNU Institute of Brain and Cognitive Science, NYU Shanghai, Shanghai, China

**Keywords:** amblyopia, contrast threshold, perceptual template model, temporal deficits, external noise, temporal window

## Abstract

**Purpose:**

Amblyopia affects not only spatial vision but also temporal vision. In this study, we aim to investigate temporal processing deficits in amblyopia.

**Methods:**

Twenty amblyopic patients (age: 27.0 ± 5.53 years, 15 males), and 25 normal observers (age: 25.6 ± 4.03 years, 15 males) were recruited in this study. Contrast thresholds in an orientation discrimination task in five target-mask stimulus onset asynchronies (SOA) conditions (16.7 ms, 33.4 ms, 50.0 ms, 83.4 ms, and ∞/no noise) were measured. An elaborated perceptual template model (ePTM) was fit to the behavioral data to derive the temporal profile of visual processing for each participant.

**Results:**

There were significant threshold differences between the amblyopic and normal eyes [*F*(1,43) = 10.6, *p* = 0.002] and a significant group × SOA interaction [*F*(2.75,118) = 4.98, *p* = 0.004], suggesting different temporal processing between the two groups. The ePTM fitted the data well (*χ*^2^ test, all *p*s > 0.50). Compared to the normal eye, the amblyopic eye had a lower template gain (*p* = 0.046), and a temporal window with lower peak and broader width (all *p*s < 0.05). No significant correlation was found between the observed temporal deficits and visual acuity in amblyopia (*p*s > 0.50). Similar results were found in the anisometropic amblyopia subgroup. No significant difference was found between the fellow eyes of the monocular amblyopia and the normal eyes.

**Conclusion:**

Amblyopia is less efficient in processing dynamic visual stimuli. The temporal deficits in amblyopia, represented by a flattened temporal window, are likely independent of spatial vision deficits.

## Introduction

Amblyopia, also called “lazy eye,” is a developmental disorder of the visual system caused by abnormal visual experience during early life (Kiorpes, 2019). It is one of the most common causes of vision loss in children, affecting approximately 2–5% of children worldwide (Webber and Wood, 2005; Wang et al., 2011; Tailor et al., 2016; Faghihi et al., 2017). Amblyopia can induce structural and functional changes in the visual pathways, which are commonly believed to begin at the level of the primary visual cortex (V1) (Kiorpes, 2019). Typically, patients with amblyopia exhibit deficits including reduced spatial contrast sensitivity (Hess and Howell, 1977; Kosovicheva et al., 2019), loss of stereopsis (Giaschi et al., 2013; Levi et al., 2015), reduced grating and Vernier acuity (Levi and Klein, 1982, 1985; Birch and Swanson, 2000), spatial distortions (Barrett et al., 2003; Piano et al., 2015), deficits in spatial localization (Hess and Holliday, 1992), and spatial localization deficit (Hess and Holliday, 1992). Amblyopia also accompanied temporal deficits, such as reduced temporal contrast sensitivity (Levi and Harwerth, 1977; Wesson and Loop, 1982; Kosovicheva et al., 2019), higher flicker fusion frequency (Miles, 1949; Manny and Levi, 1982), as well as deficits in local and global motion (Simmers et al., 2003, 2006; Ho et al., 2005; Ho and Giaschi, 2006, 2007). Moreover, accumulating evidences suggested that the temporal deficit could be independent from the spatial deficit in amblyopia (Kiorpes et al., 2006; Huang et al., 2012; Tao et al., 2019).

Extracting ecologically relevant information in a complex environment is essential for humans. The perceptual system must quickly pick up the signal-of-interest buried in the often noisy spatiotemporal input. In the spatial domain, many studies have shown that the amblyopic visual system is susceptible to the disturbance of irrelevant spatial information. External noise analysis provided a powerful tool to separate the observer’s ability from her intrinsic noise (Lu and Dosher, 1998, 2008, 2013; Pelli and Farell, 1999). External noise studies on amblyopia revealed that, in addition to the larger internal noise due to a shift in spatial scale of visual processing, the amblyopic eye exhibited lower processing efficiency, suggesting that it was more difficult for amblyopes to exclude external noise in the spatial domain (Wang et al., 1998; Pelli et al., 2004; Xu et al., 2006). The lower processing efficiency has been conceptualized as a “poor spatial template” of amblyopia in a perceptual template model analysis (Xu et al., 2006). By investigating the performance of amblyopic patients in the detection and position identification tasks of a bar-like stimulus using the classification image technique (Eckstein and Ahumada, 2002), Levi and Klein (2003) also concluded that the loss of efficiency in amblyopia could be partially attributed to a poor spatial template. Other studies have also found that the amblyopic visual system had a larger interaction zone in which performance on the central target is impaired by flanking stimuli (Hess et al., 2001; Levi et al., 2002).

On the other hand, how amblyopia affects patients’ ability to extract signal in dynamic visual stimuli has not been well understood. Bonneh et al. (2007) found that, in a digit identification task with the rapid serial visual presentation (RSVP) task, the size threshold difference between the fast (5 Hz) and slow (2.5 Hz) conditions was much greater in the strabismic amblyopia group than the normal group, and the threshold difference was independent of visual acuity. Their results suggest that it was more difficult for the strabismic amblyopes to identify the target embedded in a temporally crowded RSVP stream. By measuring the effect of metacontrast masking as a function of stimulus onset asynchrony (SOA) in the amblyopic and normal observers, Tytla and Steinbach (1984) found that the range of SOA for inducing masking effects with the same masks was wider in amblyopic than normal vision. Although the authors suggested that the amblyopic eye was worse in discounting distractors in time, the metacontrast mask effect was measured with a ring stimulus surrounding the target and may reflect abnormal spatial interaction in amblyopia.

In order to investigate how amblyopia affects visual processing in the temporal domain, we measured the contrast thresholds of amblyopic and normal observers in an orientation identification task with external noise masks under multiple target-mask SOA conditions, and without external noise masks. By systematically manipulating the SOA of the external noise masks relative to the target, we quantified masking effects at different SOA conditions and used the results to infer how processing efficiency changes as a function of time based on the perceptual template model (PTM) (Lu and Dosher, 1999, 2008). The original PTM has been applied in studying the spatial template in amblyopia (Xu et al., 2006). The elaborated PTM (ePTM) with additional parameters describing the temporal profile of visual processing has been used to study effect of attention (Lu et al., 2004) and aging (He et al., 2020). Here, we adopted the ePTM (Lu and Dosher, 1999, 2008; Lu et al., 2004; Hou et al., 2014) to fit the trial-by-trial response data for each observer. The temporal profile of the perceptual template was estimated based on the best fitting model parameters and compared between the amblyopic and normal groups.

## Materials and Methods

### Participants

Twenty amblyopic patients (age: 27.0 ± 5.53 years; 15 males), and 25 age-matched normal observers (age: 25.6 ± 4.03 years, 15 males) participated in the study. All participants went through detailed ophthalmological examinations. The observers in the normal group had normal or corrected-to-normal vision (visual acuity, VA ≤ 0.0 logMAR). Eye dominance was determined with the hole-in-card method for the normal group. Among the 20 amblyopic participants, there were 16 participants with monocular amblyopia (A1 – A16) and four with binocular amblyopia (A17 – A20). These participants could also be classified as anisometropic amblyope (A1 – A13, A18 – A20, *n* = 16), combined strabismic-anisometropic amblyope (combined with anisometropia, A14 – A15, *n* = 2), and deprivation amblyope (A16 – A17, *n* = 2). The detailed clinical information of all amblyopic participants was listed in Supplementary Table 1.

All observers were naive about the purpose of the study and provided written informed consent. Most of the amblyopic observers had the experience of psychophysical experiments before. The study adhered to the tenets of the Declaration of Helsinki and was approved by the institutional review board of human subject research of the Eye Hospital, Wenzhou Medical University.

### Apparatus

The experiment was conducted in a dimly lighted room. We used customized programs to present visual stimuli and collect responses from the observers. The programs used in the experiment were coded in MATLAB (The MathWorks Inc., Natick, MA, United States) with Psychtoolbox extensions (Kleiner et al., 2007), and run on a HP ProDesk 680 G2 MT computer (Hewlett-Packard, Palo Alto, CA, United States). Stimuli were displayed on a gamma-corrected Sony Multiscan G520 CRT display (Sony Corp., Tokyo, Japan) with a mean luminance of 44.6 cd/m2. The resolution of the display was 800 × 600 pixels and the refresh rate was 120 Hz. The viewing distance was 1.44 m, at which each pixel subtended 0.01 degrees. A chin rest was used to minimize head movement during the experiment. Observers viewed the stimuli monocularly with their best correction (if any). The non-tested eye was occluded by an opaque patch.

### Stimuli

**FIGURE 1:**
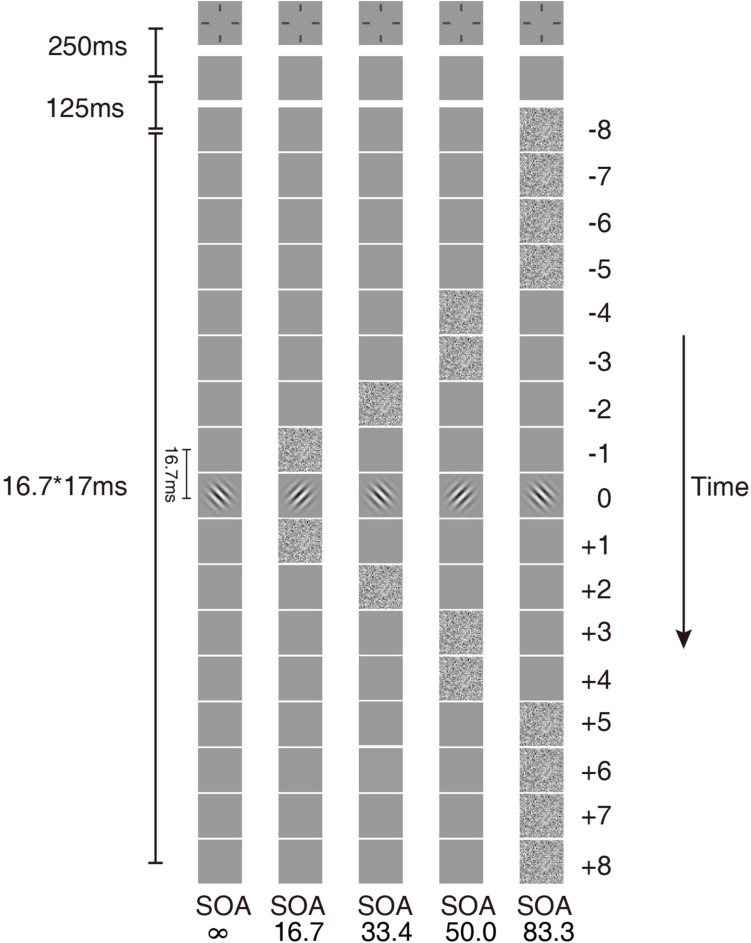
Temporal configurations of the five masking conditions in the experiment. In condition SOA ∞, only the Gabor target was presented. In the other four conditions, the external noise masks were presented symmetrically around the target.

The stimulus in each trial consisted of a sequence of 17 image frames. Each image frame lasted two display refresh cycles (16.7 ms) (Figure 1). The image in the ninth frame was the target, a Gabor oriented +45° or −45° from vertical with a center spatial frequency of 1 cycle per degree (cpd). The size of the Gabor was 300 × 300 pixels. The standard deviation of the Gaussian window was the same as the wavelength of the Gabor.

The external noise masks were white noise images, in which the contrast of each pixel was independently sampled from a Gaussian distribution with a mean of 0 and a standard deviation of 0.333. The size of the external noise images was also 300 × 300 pixels. The size of each noise element was 10 × 10 pixels, which was one-fifth of the wavelength of the signal stimuli. All signal and external noise frames were centered at fixation. The external noise frames were always symmetric around the signal frame. For simplicity of description, the frames in the sequence were numbered from −8 to 8, with the signal frame at 0. Four external noise configurations were used: external noise images occurred in ±1, ±2, ±3 ∼±4, or ±5 ∼±8 frames, which in the rest of the article are noted as SOA 16.7 ms, 33.4 ms, 50.0 ms, and 83.4 ms, respectively (Figure 1). There was also a condition with no noise mask (SOA ∞, Figure 1).

The remaining frames in the 17-frame sequence other than those of signal or external noise were filled with blank frames with background luminance. The same temporal configurations were also used in Lu et al. (2004) and He et al. (2020).

### Design

The quick forced-choice method (Lesmes et al., 2015) targeted at three different performance levels (percentage correct = 65, 75, and 85%, respectively) was used to measure contrast thresholds. Data collected at three different performance levels are necessary to constrain the non-linearities in the PTM (Lu and Dosher, 1999, 2008). Trials of the five temporal masking conditions were interleaved randomly with equal number of trials in each test session. Each test session consisted of 750 trials and lasted about 40 min. The two eyes of amblyopic patients were measured separately. Because no significant difference between the two eyes of normal observers was found in our previous study (He et al., 2020), only the dominant eye of the normal observer was tested. Each observer was given a practice session of about 75 trials before the experiment started.

### Procedure

Each trial began with a brief tone. At the same time, a fixation crosshair was presented in the center of the screen and lasted 250 ms. A blank screen (125 ms) with background luminance followed. Then the 17-frame (16.7 × 17 = 283.9 ms) stimulus sequence was presented and ended with another blank frame. Observers were instructed to indicate whether the Gabor stimulus was oriented to the left or to the right from vertical by pressing the left or right arrow key on the keyboard. Auditory feedback was provided after each correct response. A new trial started 500 ms after the response was made.

### Analysis

We first calculated the contrast threshold in different SOA conditions for each observer. The raw response data were pooled across performance levels in each condition and fit with the Weibull function using a maximum likelihood procedure (Watson, 1979). The threshold from the best fitting model was used to analyze masking effects. To quantitatively estimate the characteristics of temporal processing, the ePTM (Lu et al., 2004) was fit to the data (see “The Model” section below).

Repeated measures ANOVA and *t*-test were used to compare the thresholds and parameters between the amblyopic and normal groups. Degrees of freedom were corrected using Greenhouse-Geisser estimates of sphericity in the case of violation of sphericity. Comparisons were made between the amblyopic eyes (AE, *n* = 20) and normal eyes (NE, *n* = 25), the amblyopic eyes of the anisometropic amblyopia subgroup (AAE, *n* = 16) and the NE, as well as the fellow eyes of the monocular amblyopia subgroup (FE, *n* = 16) and the NE. Please note the AAE were a subset of the AE. For binocular amblyopic observers, the weaker eye (with worse VA) was used as the amblyopic eye except observer A19. The left eye of A19 was used as the amblyopic eye because the data from his weaker (right) eye was excluded based on the result from the ePTM analysis – the full width at half maximum (FWHM) of the temporal window of A19’s weaker eye exceeded two standard deviations from the means of the amblyopic group.

### The Model

**FIGURE 2:**
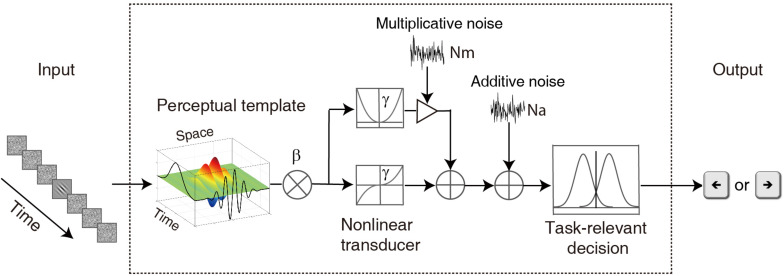
A diagram of the elaborated perceptual template model (ePTM). The visual information first passes through a perceptual template. The perceptual template has a total signal gain of *β*. It also has different weight at different time. After the template matching, the information goes through a non-linear transducer with an exponent of *γ*, then gets contaminated by an internal additive noise *Na* and a multiplicative noise *Nm*, and finally is sent to the decision unit. The temporal weights at different SOAs can be inferred from the masking effects at different target-mask SOA conditions.

To characterize the temporal properties of visual processing in the amblyopic and normal groups, we adopted the ePTM (Lu et al., 2004) to analyze the behavioral data and to derive the temporal processing profile. The key idea of the ePTM is that masking effects at different SOA conditions represent the relative impacts of the external noise mask, and can be used to infer the temporal profile of the template, which has different weights *W*_*t*_ at different time *t* (from −8 to 8) for the 17-frame stimuli (Figure 2). The external noise masks were presented at ±1, ±2, ±3 ∼±4, or ±5 ∼±8 frames symmetrically around the signal frame. That is to say, we can only obtain the average weight in the multi-frame external noise conditions.

(1) $$W_{t}=\left\{ \begin{array}{c} W_{16.7},i\,f\,t=-1,1,\\ W_{33.4},i\,f\,t=-2,2,\\ W_{50.0},i\,f\,t=-4,-3,3,4,\\ W_{83.4},i\,f\,t=-8,-7,-6,-5,5,6,7,8. \end{array}\right.$$

Because the total gain of the perceptual template to external noise is normalized to 1.0 in the PTM (Lu and Dosher, 1999), the weights of the perceptual temporal window satisfies the constraint:

(2a) $$\sum_{t=-8}^{8}W_{t}^{2}=1.$$

It can also be written as:

(2b) $$\sum_{i=1}^{4}F_{i}\,W_{i}^{2}=1.$$

where *F*_*i*_ = 2, 2, 4, and 8, *F*_*i*_ represents the number of frames, *i* = 1, 2, 3, and 4 corresponds to condition SOA 16.7 ms, 33.4 ms, 50.0 ms, and 83.4 ms, respectively. For external noise images each with rms contrast σ, the total variance of external noise in a given temporal configuration is:

(3) $$N_{e\,x\,t}^{2}=\sum_{t=-8}^{8}{\left(W_{t}\,\sigma_{t}\right)}^{2}=\sum_{i=1}^{4}F_{i}\,{\left(W_{i}\,\sigma \right)}^{2}.\;$$

where σ_*t*_ = σ, when the external noise frames were presented and σ_*t*_ = 0, when there was no mask (Figure 1).

Combine the equation 3 with the original PTM (Lu and Dosher, 1999, 2008), we have:

(4) $$d^{\prime }=\frac{{\left(\beta \,c\right)}^{\gamma }}{\sqrt{\left({\left(\beta \,c\right)}^{2\,\gamma }+{\left(\sum_{t=-8}^{8}{\left(W_{t}\,\sigma_{t}\right)}^{2}\right)}^{\gamma }\right)\,N\,m^{2}+N\,a^{2}}}.$$

where *Na*, *Nm*, *β*, and *γ* represent additive internal noise, multiplicative noise, overall gain of the perceptual template, and non-linear transducer function of the system, respectively (Lu and Dosher, 1999, 2008).

The probability of making a correct response in a trial can be derived from the *d’* equation for each observer (Hacker and Ratcliff, 1979):

(5) $$P\,\left(c\right)=\int_{-\infty }^{+\infty }\phi \,\left(x-d^{\prime }\,\left(c\right)\right)\,{\text{Φ}}^{m-1}\,\left(x\right)\,d\,x.$$

where, *m* = 2 for the 2-alternative forced orientation identification task, and, ϕ() and Φ() are the probability density and cumulative probability density functions of a standard normal distribution, respectively. The ePTM had seven free parameters: Na, Nm, *β*, *γ*, *W*_16.7_, *W*_33.4_, and *W*_50.0_. Weight *W*_83.4_ was calculated from equation 2 based on the values of the other three weights. The model was fit to raw response data in each trial for each observer using a maximum likelihood procedure.

## Results

### Contrast Thresholds in Amblyopia

**FIGURE 3:**
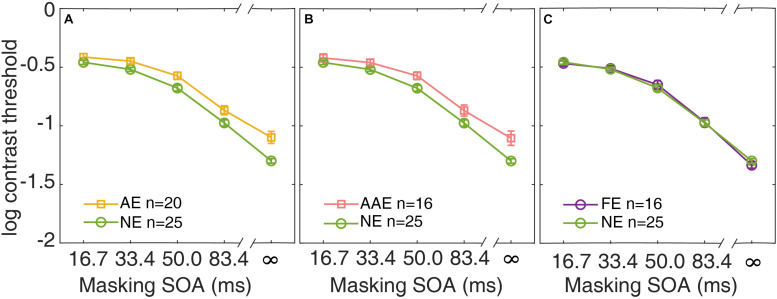
Average contrast thresholds as a function of SOA are shown for the AE **(A)**, the AAE **(B)**, and the FE **(C)**. The average contrast thresholds of the NE are also plotted in each panel for comparison. Yellow: AE. Pink: AAE. Purple: FE. Green: NE. Error bars represent ±1 standard error.

In Figure 3A, the average contrast thresholds of the AE and NE are plotted as functions of SOA. A two-way repeated measures ANOVA with factors of group and SOA was conducted. Both factors group and SOA were found to have significant effects on threshold [group: *F*(1,43) = 10.6, *p* = 0.002; SOA: *F*(2.75,118) = 597, *p* = 3.20 × 10^–69^]. The contrast thresholds were higher in the AE than the NE. There was also a significant interaction between the two factors [*F*(2.75,118) = 4.98, *p* = 0.004]. It indicated that the pattern of the masking effect over different SOAs was different between the AE and NE.

Similar analysis was applied to the subgroup of the patients with anisometropic amblyopia. The average contrast thresholds of the AAE are plotted against SOA, along with that of the NE in Figure 3B. A two-way repeated measures ANOVA showed that both group and SOA had significant effects [group: *F*(1,39) = 8.14, *p* = 0.007; SOA: *F*(2.72,106) = 544, *p* = 2.42 × 10^–62^]. The patients with anisometropic amblyopia had higher contrast thresholds than the normal participants. The interaction between group and SOA was also significant [*F*(2.72,106) = 4.77, *p* = 0.005].

The data from the fellow eyes of the subgroup of patients with monocular amblyopia was also analyzed. The average contrast thresholds of the FE and the NE are plotted as functions of SOA in Figure 3C. There was no significant difference in threshold between the groups [*F*(1,39) = 0.003, *p* = 0.96]. The group × SOA interaction was not significant [*F*(2.91,13.6) = 0.90, *p* = 0.44]. The fellow eyes of the patients with monocular amblyopia had comparable contrast thresholds as the normal eyes.

### The Results of Model Fitting

**FIGURE 4:**
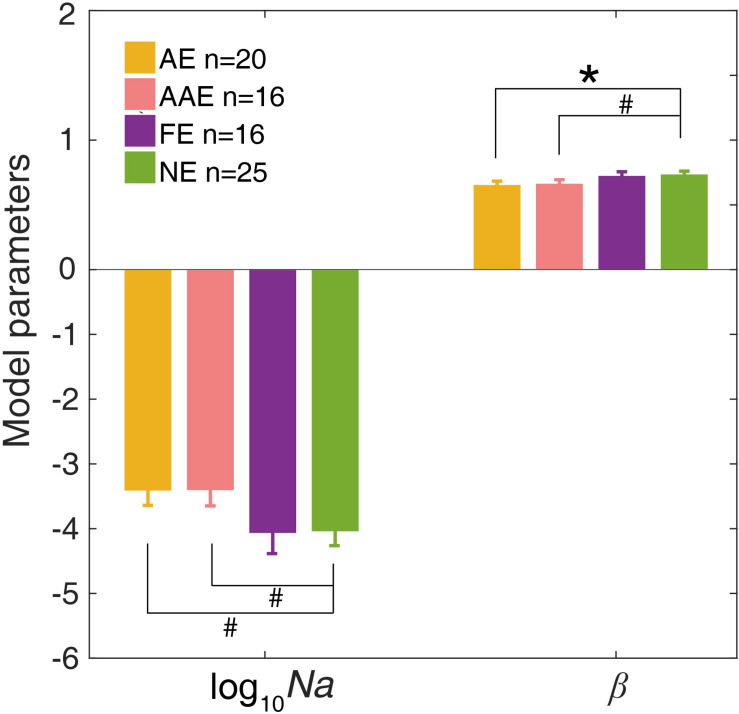
The average *Na* (in log10 units) and *β* of the best-fitting ePTM. Yellow: AE. Pink: AAE. Purple: FE. Green: NE. Error bars represent ±1 standard error. *: *p* < 0.05. #: 0.05 < *p* < 0.1.

The ePTM provided good fits to the raw response data for all participants (*χ*^2^ test, all *ps* > 0.50). We first looked at the internal additive noise *Na* and template gain *β* of the best fitting ePTM (Figure 4). There was a marginally significant difference in *Na* between the AE and NE [−3.40 ± 1.03 vs. −4.03 ± 1.17, *t*(43) = 1.89, *p* = 0.066]. There was also a marginally significant difference in *Na* between the AAE and NE [−3.40 ± 0.98 vs. −4.03 ± 1.17, *t*(39) = 1.81, *p* = 0.078]. No significant *Na* difference was found between the FE and NE [*t*(39) = −0.062, *p* = 0.95]. The template gain *β* in the AE was significantly smaller than that in the NE [0.65 ± 0.13 vs. 0.73 ± 0.13, *t*(43) = −2.06, *p* = 0.046]. The *β* in the AAE was marginally smaller than that in the NE [0.66 ± 0.13 vs. 0.73 ± 0.13, *t*(39) = −1.74, *p* = 0.090]. There was no significant *β* difference between the FE and NE [*t*(39) = −0.28, *p* = 0.78]. No significant difference in the multiplicative noise *Nm*, or the non-linear exponent *γ* was found in any comparisons (all *p*s > 0.10).

### Change in Temporal Window

**FIGURE 5:**
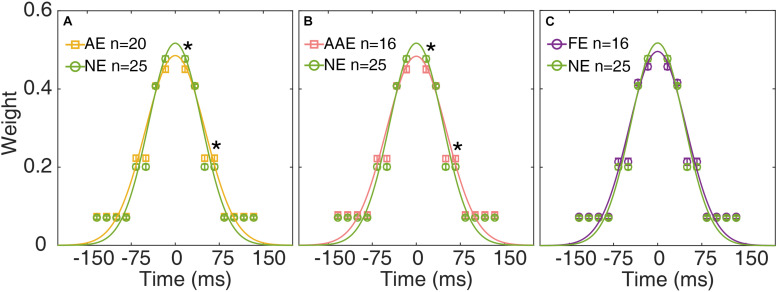
The average temporal weights of the best-fitting ePTM of the AE (yellow), AAE (pink), and FE (purple) are plotted as functions of time in **(A–C)**, respectively. The average temporal profile of the NE (green) is also plotted in each panel. *Error bar*: ±1 standard error. *: *p* < 0.05. The continuous curves are the best-fitting Gaussians.

The temporal weights *W*_16.7_, *W*_33.4_, *W*_50.0_, and *W*_83.4_ of the AE, FE, and NE were derived from the best fitting ePTM. The temporal weights *W*_*t*_ over the range (−8 to 8 frames) can be derived from *W*_16.7_, *W*_33.4_, *W*_50.0_, and *W*_83.4_ based on equation 1. The average temporal profile of the AE, AAE, and FE are plotted as functions of time in Figures 5A–C, respectively. The unit of the abscissa has been converted into the actual time (ms). The average temporal profile of the NE is also plotted in each panel.

A repeated measures ANOVA was applied to the weights at different SOAs. The effect of group was not significant [*F*(1,43) = 0.13, *p* = 0.72]. There was a significant effect of SOA [*F*(2.13,91.5) = 1068, *p* = 2.31 × 10^–65^]. There was also a significant interaction between group and SOA [*F*(2.13,91.5) = 3.29, *p* = 0.039], suggesting that the temporal profiles were different between the AE and NE. *Post-hoc* analysis showed that the temporal weight at SOA 16.7 ms was significantly lower in the AE group than in the NE group [one-tailed, *t*(41.0) = −2.41, *p* = 0.010. Figure 5A], the temporal weight at SOA 50.0 ms was higher in the AE group than in the NE group [one-tailed, *t*(43) = 2.12, *p* = 0.020]. There was no significant weight difference at SOA 33.4 and 83.4 ms (all *p*s > 0.20).

We then compared the temporal weights between the AAE and NE. Similarly, there was no significant weight difference between the two groups [*F*(1,39) = 0.013, *p* = 0.91]. There was a significant effect of SOA [*F*(2.16,84.1) = 984, *p* = 2.90 × 10^–60^]. There was also a significant interaction between group and SOA [*F*(2.16,84.1) = 3.05, *p* = 0.032]. The temporal weight at SOA 16.7 ms was significantly lower in the AAE than in the NE group [one-tailed, *t*(39) = −2.32, *p* = 0.013], the temporal weight at SOA 50.0 ms was higher in the AE than in the NE [one-tailed, *t*(39) = 1.99, *p* = 0.027]. There was no significant weight difference at SOA 33.4 and 83.4 ms (all *p*s > 0.20).

When comparing the weights between the FE and NE, an ANOVA revealed that only SOA had significant effect [*F*(2.05,79.9) = 1154, *p* = 4.08 × 10^–60^]. No significant weight difference was found between the FE and NE [*F*(1,39) = 1.18, *p* = 0.29]. The interaction between group and SOA was not significant [*F*(2.05,79.9) = 1.90, *p* = 0.16].

**FIGURE 6:**
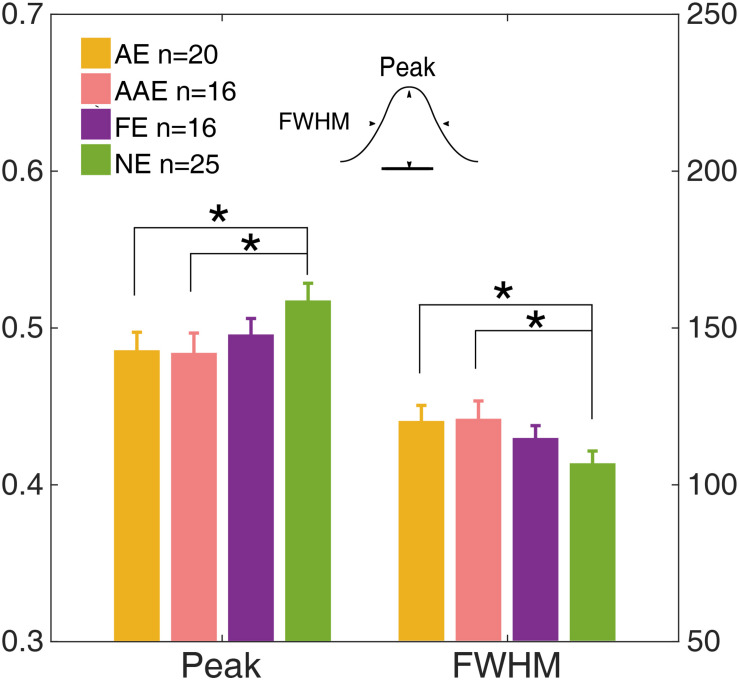
The peak and full width at half maximum (FWHM) of the temporal window of the AE (yellow), AAE (pink), FE (purple), and NE (green). The left *y*-axis shows the peak and the right *y*-axis shows the FWHM. *Error bar*: ±1 standard error. *: *p* < 0.05.

The temporal weight decreased as the external noise mask was presented further (in time) away from the onset of the target, thus the temporal profile was termed as the “temporal window.” A Gaussian function, *g*(t) = *peak* ⋅ exp (− ($\frac{t^{2}}{2\,\sigma^{2}}$)), was fit to *W*_*t*_ to quantify the shape of the temporal window. The residual of each data point was weighted to make sure that the data derived in each external noise condition contributed equally to the entire fitting. The peak amplitude and full width at half maximum (FWHM), computed as $2\,\sqrt{2\,\ln\left(2\right)}\,\sigma$, were derived for each observer (Figure 6).

The peak and FWHM of the AE, AAE, and FE are shown in Figure 6. The peak amplitude of the AE was significantly lower than that of the NE [0.49 ± 0.051 vs. 0.52 ± 0.044, *t*(43) = −2.24, *p* = 0.030]. The FWHM of the AE was significantly greater than that of the NE [120 ± 22.1 ms vs. 107 ± 15.7 ms, *t*(43) = 2.39, *p* = 0.020]. The peak amplitude of the AAE was significantly lower than that of the NE [0.48 ± 0.050 vs. 0.52 ± 0.044, *t*(39) = −2.25, *p* = 0.031]. The FWHM of the AAE was significantly greater than that of the NE [121 ± 22.6 ms vs. 107 ± 15.7 ms, *t*(39) = 2.38, *p* = 0.022]. No significant peak or FWHM difference was found between the FE and NE (all *p*s > 0.10).

### The Relationship Between the Temporal Window and Spatial Vision

**FIGURE 7:**
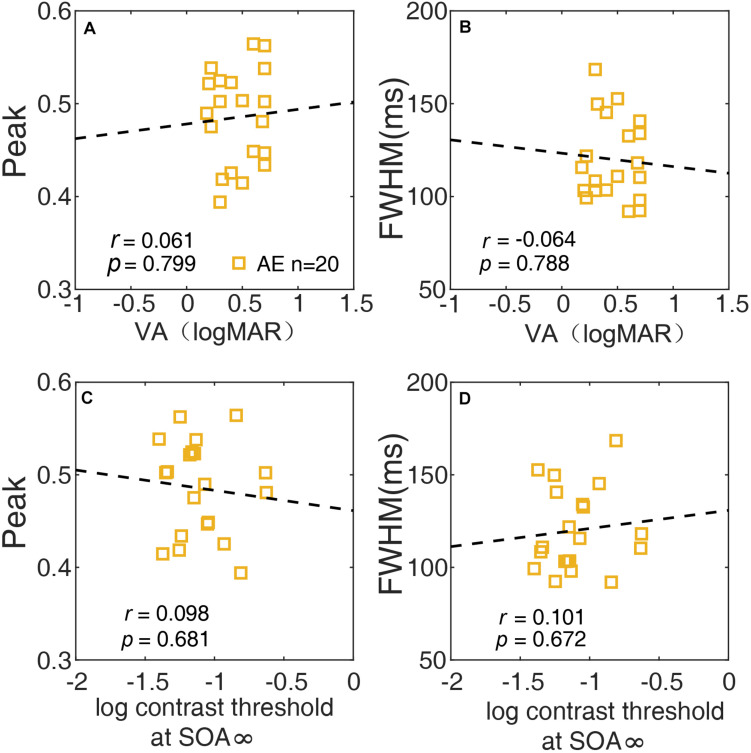
The relationship between the peak of the temporal window and VA **(A)**, between the width (FWHM) of the temporal window and VA **(B)**, between the peak of the temporal and contrast threshold at SOA ∞ **(C)**, and between the width (FWHM) of the temporal window and contrast threshold at SOA ∞ **(D)** of the AE are plotted.

We also investigated the relationship between the temporal window and spatial vision using correlation analysis of the AE. The peak and FWHM of the temporal window are plotted against the visual acuity of the AE in Figures 7A,B, respectively. Neither the correlation between the peak and visual acuity (*r* = 0.061, *p* = 0.80), nor that between the FWHM and visual acuity (*r* = −0.064, *p* = 0.79) was significant. In Figures 7C,D, the peak and FWHM are plotted against the thresholds of SOA ∞ (no noise condition) for the AE. Neither correlation was significant (all *p*s > 0.60). The results indicated that the temporal deficit in amblyopia was likely independent of spatial vision.

## Discussion

Using an orientation identification task with external noise masks in various target-mask SOA conditions, we measured the contrast thresholds of amblyopic and normal observers. The contrast thresholds of the amblyopic eyes were higher than that of the normal eyes. A significant interaction between group and SOA was found, indicating that the pattern of masking effect in the amblyopic eyes was different from that in the normal eyes. By fitting the ePTM to the trial-by-trial data, we derived several parameters of the observer model. The additive noise of the amblyopic eyes was marginally higher than that of the normal eyes, and the template gain of the amblyopic eyes was significantly lower than the normal eyes. A further analysis of the weight parameters of the temporal window revealed that the amblyopic eyes had a flatter (lower peak and broader width) temporal window than the normal eyes. No significant difference was found between the shape of the temporal window and spatial vision of the amblyopic eyes. Additional analysis showed that the anisometropic amblyopic eyes had similar temporal deficits. However, we did not observe any difference in the contrast threshold or temporal window between the fellow eyes of the patients with monocular amblyopia and the normal eyes.

The different patterns of the masking effects between the AE and NE (Figure 3A), as evidenced by the significant interaction between the group and SOA in ANOVA, indicated that the masking effect (computed as the threshold elevation) of the AE was smaller than that in the NE. This result did not mean that the AE tolerated the noise better than the NE. This is because the threshold difference between the external noise condition and the no noise condition was determined jointly by the internal additive noise and the template efficiency (external noise exclusion) (Lu and Dosher, 1999, 2008). The counterintuitive result was due to the marginally increased internal additive noise and significantly decreased template efficiency as revealed by the ePTM. Our result was consistent with that reported in Xu et al. (2006). As shown in their Figures 2, 3, the threshold difference between high noise and no noise conditions was smaller in the amblyopic eyes than that in the normal eyes. They also found that the amblyopic patient had an increased internal additive noise and a defective template.

The decreased template gain indicated that it was difficult for amblyopia to exclude external noise and therefore a lower processing efficiency in amblyopia (Wang et al., 1998; Pelli et al., 2004; Xu et al., 2006). By systematically manipulating the SOA of the noise masks, we can estimate the temporal profile of the perceptual template (temporal window), which represents how the processing efficiency of the visual template varies in time. We found that the temporal window of the amblyopic visual processing was flattened relative to the normal one. It should be noted that the temporal window applies to both signal and noise. If the temporal window is flat, it means the processing efficiency at small SOA (i.e., 16.7 ms) is low – it is true that the template excluded more external noises, but it also attenuated more signals. Moreover, the broader extent of the temporal window also implies that the amblyopic eyes are not properly tuned to the timing of signal thus have more difficulties in processing dynamic visual information, even at low spatial frequencies. Taken together, our finding combined with the previous studies in spatial domain suggests the amblyopia has an impaired spatio-temporal template.

Amblyopia is commonly associated with strabismus or anisometropia in early life. Apart from the oculomotor difference, it has been shown that the pattern of visual deficits were different between strabismic and anisometropic amblyopes (McKee et al., 2003). Using a RSVP digit identification task, Bonneh et al. (2007) found that strabismic amblyopes showed a significant VA reduction in the fast RSVP condition. However, the anisometropic amblyopes and normal observers did not show any significant VA difference between the fast and slow conditions. In our study, with a more quantitative approach, we found that anisometropic amblyopia had a temporal window with lower peak and broader width, indicating that anisometropic amblyopia also led to temporal deficits. Although there were not enough strabismic participants in the current experiment for us to draw any concrete conclusion on how the temporal window in strabismic amblyopia was affected, we performed some preliminary analyses based on the data from the two combined strabismic-anisometropic amblyopes. Similar to the anisometropic eye, the combined strabismic- anisometropic eyes showed significantly different results compared to the normal eyes. No significant difference between the combined strabismic- anisometropic and anisometropic eyes was found. The detailed result was described in the Supplementary Material: Preliminary Results in Strabismic Amblyopia. It is possible that the temporal deficits in anisometropic amblyopia are less severe than those in strabismic amblyopia. Future studies are necessary to evaluate whether subtypes of amblyopia have different temporal processing deficits.

The defective temporal window in amblyopia could be due to the deficits in low level visual processing. In an animal study, Kiorpes et al. (2006) measured sensitivity to visual motion in random dot displays for strabismic and anisometropic amblyopic monkeys. They found that amblyopic losses in motion sensitivity were not correlated with losses in spatial contrast sensitivity, and also found a specific impairment for detecting long temporal offsets, revealing a deficit in spatiotemporal integration in amblyopia which cannot be explained by the lower spatial resolution of amblyopic vision. Similarity, in human studies, Spang and Fahle (2009) used a time-based figure–ground segregation task and demonstrated that the temporal resolution of the amblyopic eye was reduced. Huang et al. (2012) and Tao et al. (2019) found that the temporal synchrony sensitivity of amblyopic eyes was higher than that of the fellow eyes, which was uncorrelated with the visual acuity, suggesting that amblyopes have a low-level temporal processing deficit in the fovea.

Some studies have suggested that abnormal visual experience during the developmental critical period in amblyopia could also affect higher level of cortical areas (Popple and Levi, 2008; Farzin and Norcia, 2011; Perdziak et al., 2018). Popple and Levi (2008) found that, when viewing displays with their amblyopic eyes, observers had a shallower attentional blink 200 ms after the first target, compared with the preferred eye, depending on the depth of amblyopia, and made more wrong responses consisting of non-distractor letters, when the distractors and targets were confusable. These findings may be the result of an altered time course of attention in amblyopia. Bonneh et al. (2007) also suggested that their findings reflected a lower attentional resolution, i.e., the ability to isolate successive stimuli in time. Farzin and Norcia (2011) measured the response time and accuracy of amblyopes in a variant of the Eriksen flanker task. They found a selective deficit in visual decision making when individuals with amblyopia used either the amblyopic or non-amblyopic (dominant) eye. Thus the defective temporal window found in this study could also be due to abnormalities in higher cortical areas.

Taken together, our results showed that amblyopia had temporal processing deficits captured by a flattened temporal window of visual processing, and the deficits were independent of spatial vision. How temporal deficits interact with spatial deficits, and how they affect the quality of life of amblyopic patients remain to be investigated in future studies.

## Data Availability Statement

The raw data supporting the conclusions of this article will be made available by the authors, without undue reservation.

## Ethics Statement

The studies involving human participants were reviewed and approved by the institutional review board of human subject research of the Eye Hospital, Wenzhou Medical University. The patients/participants provided their written informed consent to participate in this study.

## Author Contributions

FH, FL, Z-LL, and RC conceived the experiments. XHu, YQ, XY, and JY performed the experiments. XHu, XR, YQ, XY, JY, and XHe analyzed the data and interpreted the data. XHu, FL, Z-LL, and FH wrote the manuscript. All authors contributed to manuscript revision, read and approved the submitted version.

## Conflict of Interest

RC was employed by the company Biosysen Ltd., Shenzhen, Guangdong, China. The remaining authors declare that the research was conducted in the absence of any commercial or financial relationships that could be construed as a potential conflict of interest.
