# Supplementary material for: Temporal Characteristics of Visual Processing in Amblyopia
Source: Front Neurosci. 2021 Jun 3;15:673491. doi: 10.3389/fnins.2021.673491 (PMC8211088; doi:10.3389/fnins.2021.673491)
Supplement: Supplementary file 1 [file Table_1.docx]

Supplementary Material

Table S1. Clinical Details of the Amblyopic Patients

| **Subject** | **Sex/Age** | **Monocular or**  **Binocular** | **Type** | **Refraction**  **OD,OS** | **VA (logMAR)**  **OD,OS** | **Experienced** |
| --- | --- | --- | --- | --- | --- | --- |
| A1 | M/23 | Mono | Anis | +5.00/-1.50×15 | 0.50 | Yes |
|  |  |  |  | -6.00 | -0.08 |  |
| A2 | M/28 | Mono | Anis | -1.00/-0.5×30 | -0.06 | Yes |
|  |  |  |  | +3.50/-0.75×180 | 0.20 |  |
| A3 | M/27 | Mono | Anis | +0.5 | 0.00 | Yes |
|  |  |  |  | +2.5 | 0.22 |  |
| A4 | M/23 | Mono | Anis | -3.25/-0.50×11 | -0.08 | Yes |
|  |  |  |  | +2.50/-1.50×10 | 0.22 |  |
| A5 | M/31 | Mono | Anis | +5.00/-2.25×170 | 0.68 | Yes |
|  |  |  |  | -1.50/-0.50×15 | 0.00 |  |
| A6 | M/35 | Mono | Anis | +5.50/+1.0×170 | 0.70 | Yes |
|  |  |  |  | -2.50/-0.50×90 | 0.00 |  |
| A7 | M/25 | Mono | Anis | -0.50 | 0.00 | Yes |
|  |  |  |  | +5.00/-3.00×180 | 0.70 |  |
| A8 | M/24 | Mono | Anis | -14.50/-1.00×180 | 0.40 | Yes |
|  |  |  |  | -7.5/-0.75×10 | -0.02 |  |
| A9 | M/26 | Mono | Anis | -2.00/-0.50×165 | -0.08 | Yes |
|  |  |  |  | +2.25/+1.75×80 | 0.50 |  |
| A10 | M/28 | Mono | Anis | -2.00/-0.50×165 | -0.10 | No |
|  |  |  |  | +2.25/+1.75×80 | 0.70 |  |
| A11 | F/20 | Mono | Anis | -3.75/-1.00×5 | -0.08 | No |
|  |  |  |  | -1.25/-1.00×175 | 0.30 |  |
| A12 | F/26 | Mono | Anis | plano | -0.10 | Yes |
|  |  |  |  | +1.50/-0.50×180 | 0.18 |  |
| A13 | M/21 | Mono | Anis | -4.50 | -0.04 | Yes |
|  |  |  |  | +5.50 | 0.30 |  |
| A14 | F/21 | Mono | Combined | +1.25/-1.00×5 | 0.70 | No |
|  |  |  |  | -3.75/-0.25×10 | 0.00 |  |
| A15 | M/29 | Mono | Combined | +1.00/-0.50×45 | 0.6 | No |
|  |  |  |  | plano | 0 |  |
| A16 | M/32 | Mono | Depr | -1.50/-3.00×18 | 0.6 | No |
|  |  |  |  | -3.00/-0.5×180 | -0.02 |  |
| A17 | F/27 | Bino | Depr | -2.75/-1.00×150 | 0.4 | No |
|  |  |  |  | -3.00 | 0.7 |  |
| A18 | F/34 | Bino | Anis | 0.00/-1.00×175 | 0.40 | No |
|  |  |  |  | +0.52/-2.0×175 | 0.3 |  |
| A19 | M/23 | Bino | Anis | -8.50/-3.50×180 | 0.3 | Yes |
|  |  |  |  | -7.00/-2.50×175 | 0.34 |  |
| A20 | M/45 | Bino | Anis | +7.75 | 0.22 | No |
|  |  |  |  | +6.0 | 0.32 |  |

M, male; F, female; Mono, monocular amblyopia; Bino, Binocular amblyopia; Anis, anisometropic amblyope; Combined, combined strabismic-anisometropic amblyope; Depr, deprivation amblyope; OD, right eye; OS, left eye; plano, emmetropia; VA, vision acuity.
