# Supplementary material for: Temporal Characteristics of Visual Processing in Amblyopia
Source: Front Neurosci. 2021 Jun 3;15:673491. doi: 10.3389/fnins.2021.673491 (PMC8211088; doi:10.3389/fnins.2021.673491)
Supplement: Supplementary file 2 [file Table_2.docx]

Supplementary Material

The Results of Strabismic Amblyopia

Because there were only two combined strabismic- anisometropic amblyopes in the currently study, we cannot draw any concrete conclusion about the temporal processing in strabismic amblyopia. Nevertheless, a preliminary analysis on the data from the two combined strabismic-anisometropic amblyopes was carried and the result was shown below.

- 1. Contrast thresholds in strabismic amblyopia

In Figure S1, the average contrast thresholds of the amblyopic eyes of the anisometropic amblyopia subgroup (AAE, *n* = 16), the amblyopic eyes of the combined strabismic- anisometropic amblyopia subgroup (SAE, *n* = 2) and the normal eyes (NE, *n* = 25) are plotted as functions of SOA.

A two-way repeated measures ANOVA with factors of group and SOA was conducted to compare the thresholds between SAE and NE. Both factors group and SOA were found to have significant effects on threshold (group: *F*(1, 25) = 7.48, *p* = 0.011; SOA: *F*(3.03, 75.9) = 178 *p* = 2.33 × 10^−34^). The contrast thresholds were significantly higher in the SAE than in the NE. There was no significant interaction between the two factors (*F*(3.03, 75.857) = 0.86, *p* = 0.47).

We also compared the thresholds between SAE and AAE using a two-way repeated measures ANOVA. The effect of SOA was significant effects (*F*(2.26, 36.1) = 62.9 *p* = 4.62 × 10^−13^). There was no significant threshold difference between groups (*F*(1, 16) = 0.54, *p* = 0.47), or significant interaction between group and SOA(*F*(2.26, 36.1) = 0.71, *p* = 0.51).


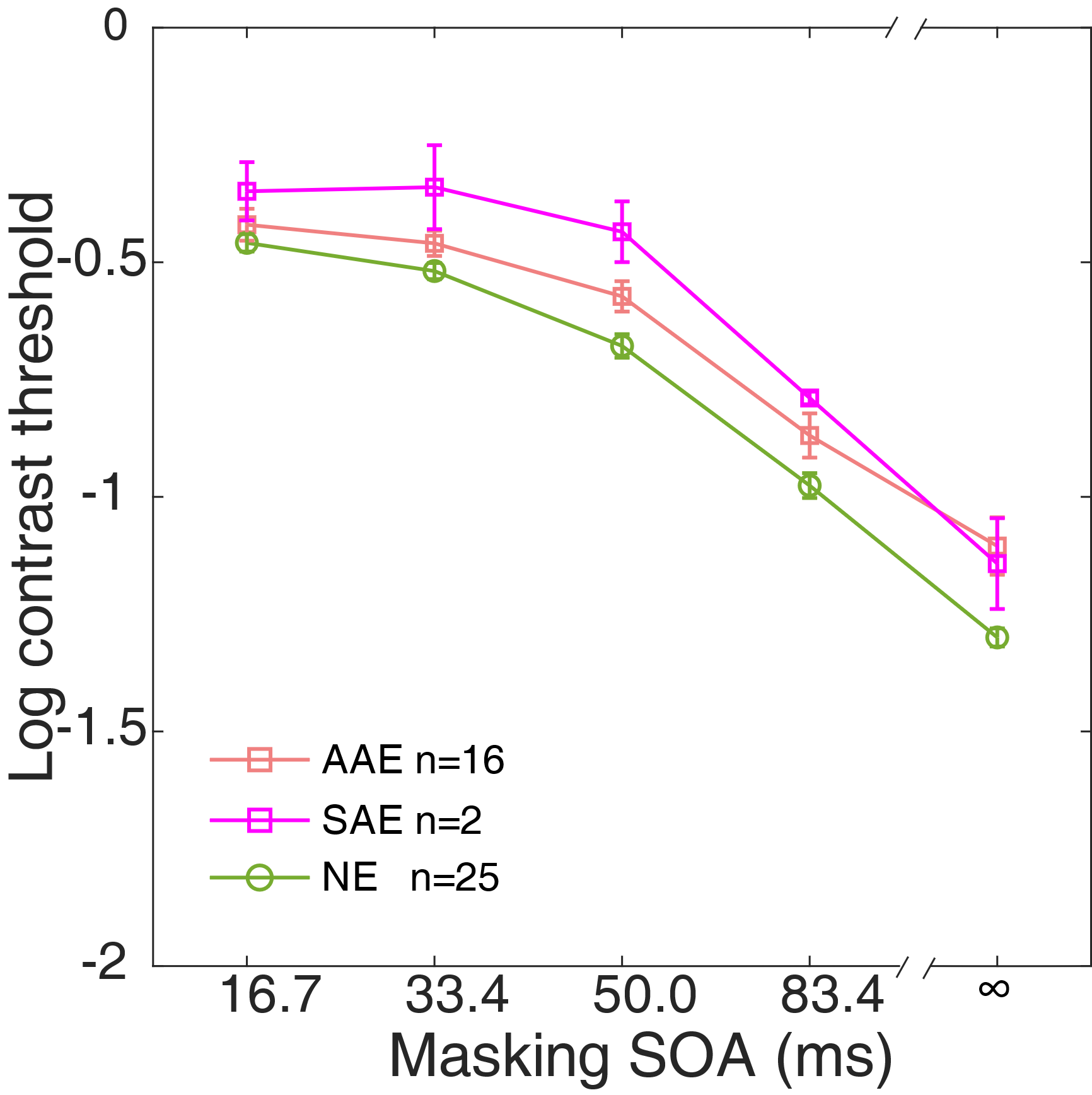


Figure S1. Average contrast thresholds as a function of SOA are shown for the AAE (Pink), the SAE (Magenta), and the NE (Green). Error bars represent ±1 standard error.

- 1. The results of model fitting

The average *Na* (in log10 units) and *β* of the best-fitting ePTM for the SAE is shown in Figure S2, along with those of the AAE and NE. There was no significant difference in *Na* between the SAE and NE (*t*(25) = −0.39, *p* = 0.70). The template gain *β* in the SAE was significantly smaller than that in the NE (0.49 ± 0.15 vs. 0.73 ± 0.13, *t*(25) = −2.42, *p* = 0.023). No significant difference between the SAE and AAE was found in *Na* (*t*(16) =1.29, *p* = 0.21), or in the *β* (*t*(16) = 1.69, *p* = 0.11). No significant difference in the multiplicative noise *Nm*, or the nonlinear exponent *γ* was found in any comparisons (all *p*s > 0.10).


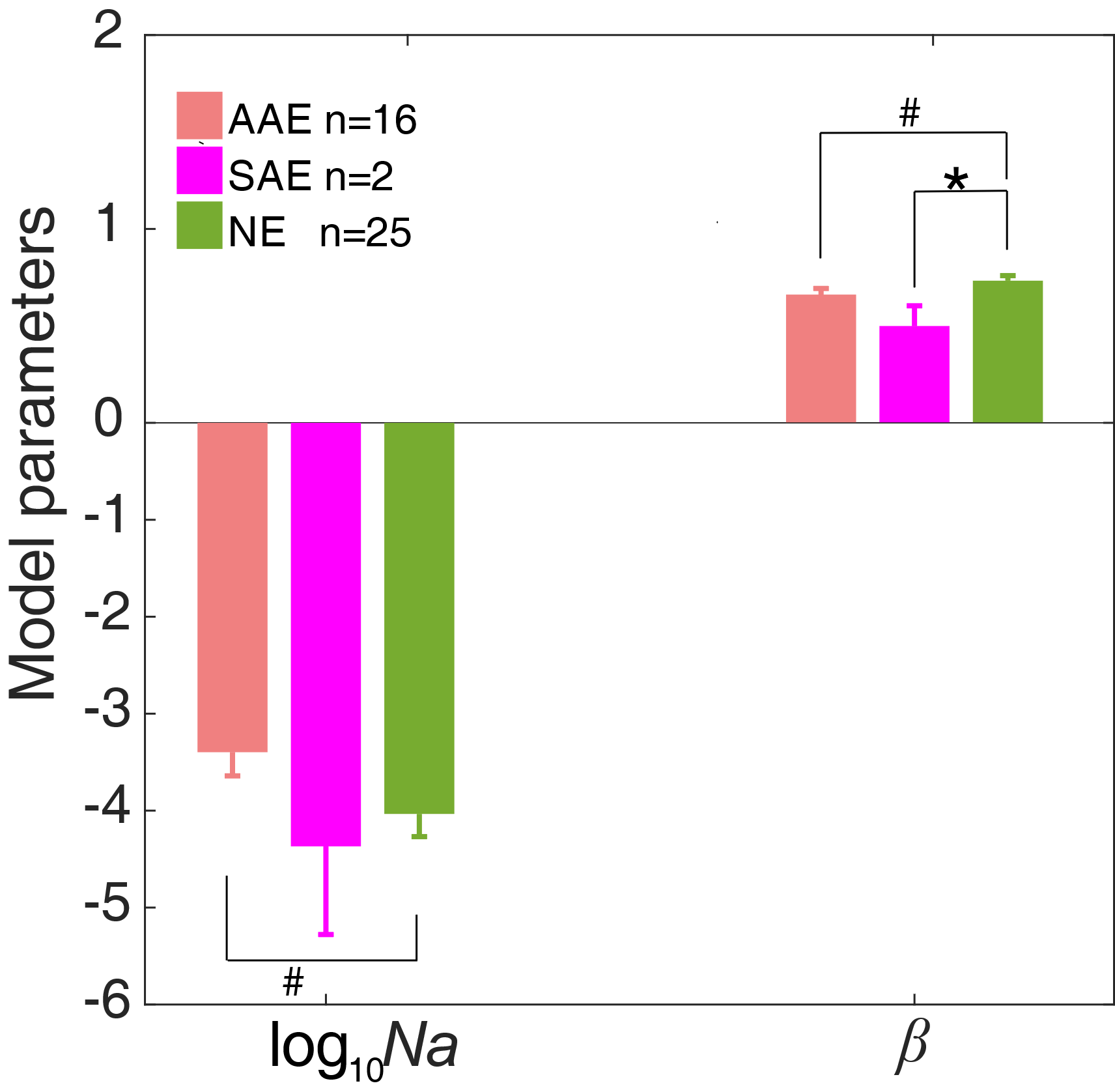


Figure S2. The average *Na* (in log10 units) and *β* of the best-fitting ePTM for the AAE, SAE and NE. Pink: AAE. Magenta: SAE. Green: NE. Error bars represent ±1 standard error. *: p < 0.05. #: 0.05 < p < 0.1.

- 1. Change in temporal window

The average temporal profile of the SAE, AAE and NE are plotted as functions of time in Figure S3. The unit of the abscissa has been converted into the actual time (ms).

A repeated measures ANOVA was used to compare the temporal weight between the SAE and NE. The effect of group was not significant (*F*(1, 25) = 0.000002, *p* = 0.999). There was a significant effect of SOA (*F*(1.96, 48.9) = 186.8, *p* = 1.72 ×10^−23^). There was also a significant interaction between group and SOA (*F*(1.96, 48.9) = 5.14, *p* = 0.01), suggesting that the temporal profiles were different between the SAE and NE. Post-hoc analysis showed that the temporal weight at SOA 16.7 ms was significantly lower in the SAE group than in the NE group (one-tailed, *t*(25) = −2.17, *p* = 0.02). The temporal weight at SOA 50.0 ms was higher in the SAE group than in the NE group (one-tailed, *t*(25) = 2.97, *p* = 0.004). There was no significant weight difference at SOA 33.4 and 83.4ms (all *p*s > 0.1).

We also compared the temporal weights between the SAE and AAE. The effect of group was not significant (*F*(1, 16) = 0.002, *p* = 0.962). There was a significant effect of SOA (*F*(1.88, 30.5) = 128, *p* = 2.69 ×10^−15^). The interaction between group and SOA was not significant (*F*(1.88, 30.5) = 1.73, *p* = 0.22), suggesting that there was no significant difference in the temporal profiles between the SAE and AAE.


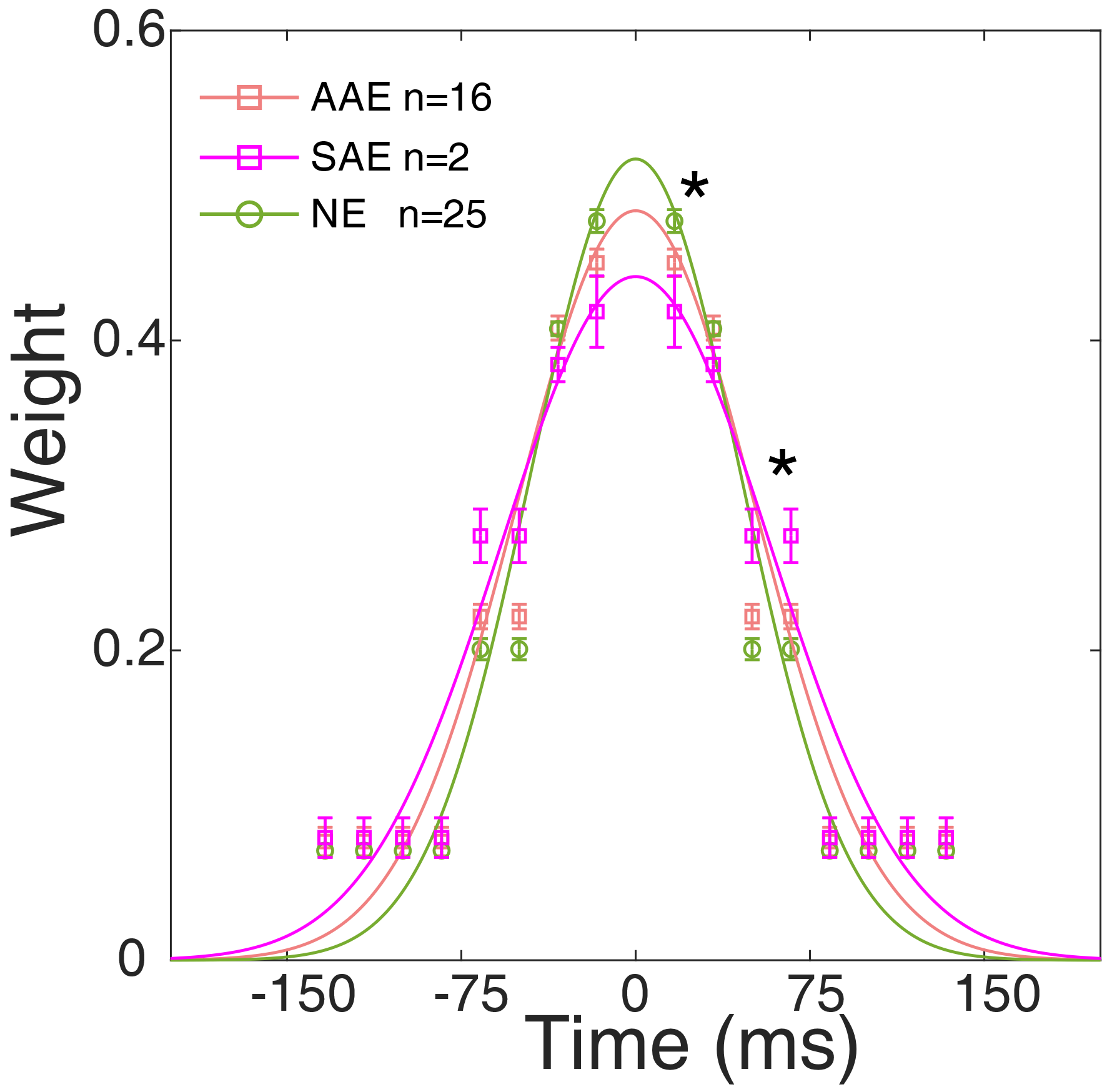


Figure S3. The average temporal weights of the best-fitting ePTM of the AAE(Pink), the SAE (Magenta), and the NE (Green) are plotted as functions of time. Error bar: ±1 standard error. *: *p* < 0.05 (SAE vs. NE). The continuous curves are the best-fitting Gaussians.

A Gaussian function was fit to the temporal weight of the SAE to quantify the shape of the temporal window. The peak and FWHM of the SAE are shown in Figure S4, along with those of AAE and NE. The peak amplitude of the SAE was significantly lower than that of the NE (0.44 ± 0.010 vs. 0.52 ± 0.044, *t*(25) = −2.41, *p* = 0.024). The FWHM of the SAE was significantly greater than that of the NE (137 ± 5.65 ms vs. 107 ± 15.7 ms, *t*(25) = 2.66, *p* = 0.013). There was no significant difference in peak amplitude between the SAE and AAE (0.44 ± 0.010 vs. 0.48 ± 0.050, *t*(16) = 1.16, *p* = 0.27), or in the FWHM (137 ± 5.65 ms vs. 121 ± 22.6 ms, *t*(16) = −0.97, *p* = 0.35).


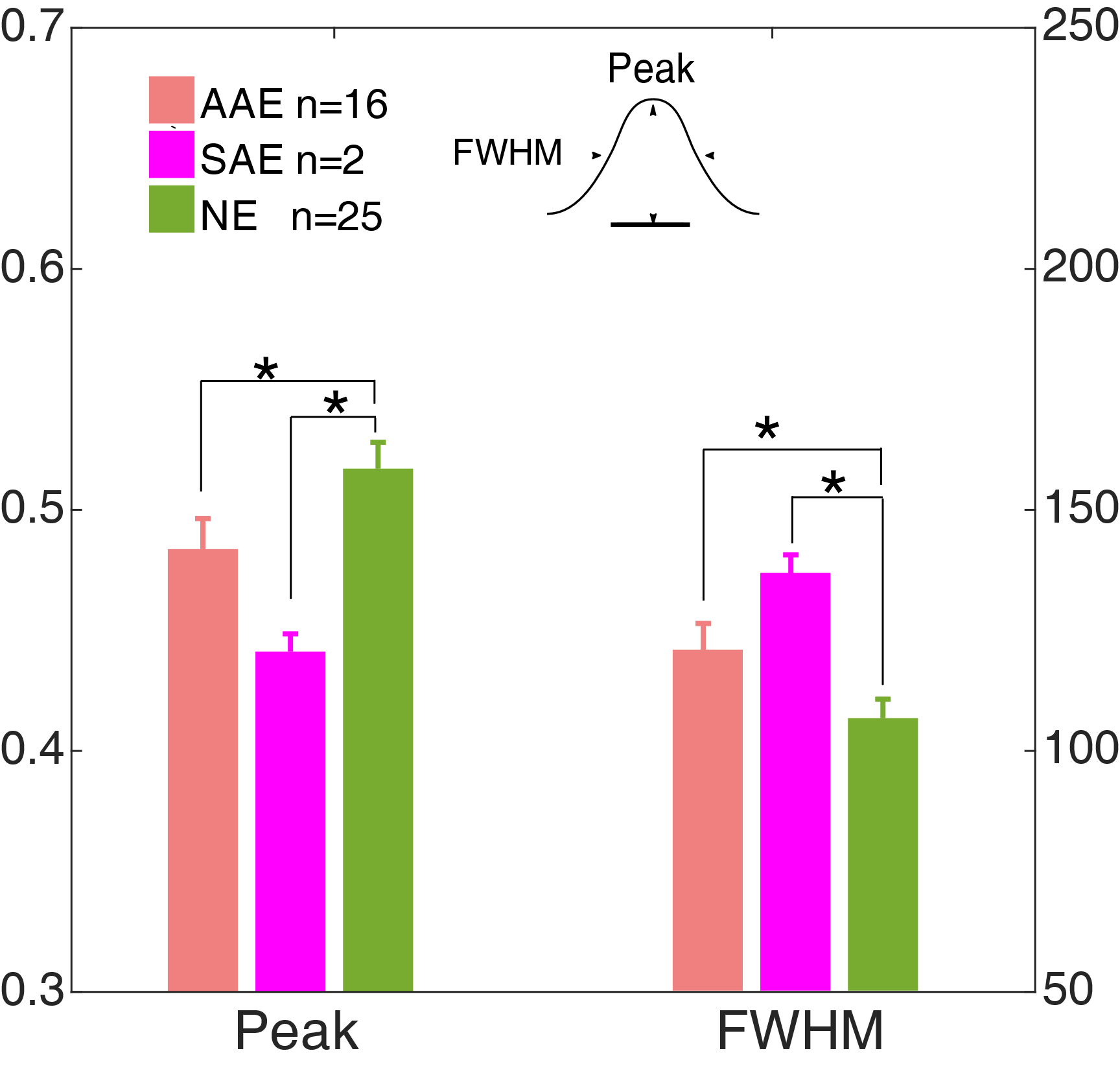


Figure S4. The peak and full width at half maximum (FWHM) of the temporal window of the AAE (Pink), the SAE (Magenta), and the NE (Green). The left y-axis shows the peak and the right y-axis shows the FWHM. Error bar: ±1 standard error. *: *p* < 0.05
